# The Prevalence of Undiagnosed Age-Related Sight-Threatening Diseases in Self-Proclaimed Healthy Individuals

**DOI:** 10.1155/2020/3709793

**Published:** 2020-11-07

**Authors:** Sophie Lemmens, João Barbosa Breda, Karel Van Keer, Tine Jacobs, Ruben Van Landeghem, Patrick De Boever, Ingeborg Stalmans

**Affiliations:** ^1^Department of Ophthalmology, University Hospitals UZ Leuven, Herestraat 49, 3000 Leuven, Belgium; ^2^Research Group Ophthalmology, Department of Neurosciences, KU Leuven, Herestraat 49, 3000 Leuven, Belgium; ^3^Health Unit, VITO (Flemish Institute for Technological Research), Boeretang 200, 2400 Mol, Belgium; ^4^Department of Ophthalmology, Centro Hospitalar Sa˜o Joa˜o, Porto, Portugal; ^5^Center of Environmental Sciences, Hasselt University, Agoralaan, 3590 Diepenbeek, Belgium; ^6^University of Antwerp, Department of Biology, Universiteitsplein, 12610 Wilrijk, Belgium

## Abstract

**Background:**

Age-related conditions such as glaucoma, age-related macular degeneration, diabetic retinopathy, and cataract have become the major cause of visual impairment and blindness in high-income countries. The aim of the current study is to investigate the prevalence of these eye diseases in a cohort of self-proclaimed healthy elderly and thus get a rough estimation of the prevalence of undiagnosed age-related eye conditions in the Belgian population.

**Methods:**

Individuals aged 55 and older without ophthalmological complaints were asked to fill in a general medical questionnaire and underwent an ophthalmological examination, which included a biomicroscopic examination, intraocular pressure measurement, axial length measurement, and acquisition of fundus pictures and optical coherence tomography scans. Information regarding follow-up was collected in those who received the advice of referral to an ophthalmologist or the advice to have more frequent follow-up visits, based on their study evaluation.

**Results:**

The cohort included 102 people and comprised 46% men (median age 70 years, range 57–85 years). Referral for additional examinations was made in 26 participants (25%). The advice to have more regular follow-up ophthalmologist visits was given to nine additional participants (9%). No significant correlations between baseline characteristics and the need for referral could be identified. Follow-up information was available for 25 out of 26 referred volunteers. Out of these, four underwent a therapeutic intervention based on study referral, up until 18 months after study participation. All four interventions took place in the age group 65–74 years.

**Conclusions:**

This study shows that, even in an elderly population with self-proclaimed healthy eyes and good general health, a significant proportion of subjects showed ocular findings that need regular follow-up and/or intervention. The frequency of prior ophthalmological examinations does not seem to be relevant to this proportion, meaning that everyone above 55 years old needs a routine ophthalmological evaluation.

## 1. Introduction

Age-related ocular diseases are the major cause of visual impairment and blindness in high-income countries and carry a major socioeconomic burden. Western Europe roughly counts one million (0.6%) blind and three to ten million (1.7–5.6%) visually impaired persons older than 40 years [1]. Age-related conditions such as cataract, age-related macular degeneration (AMD), glaucoma, and diabetic retinopathy (DRP) have become the mainstay of visual decline in the Western world and account for more than half of cases of blindness in those aged 50 or older and for 35% of cases of visual impairment in the same age group. As a single cause of visual impairment, uncorrected refractive error continues to take the lead in all age groups, worldwide as well as in high-income countries [2].

Projections made by Finger and Scholl estimate that visual impairment will affect 5–25% of an elderly population in a high-income region over a 5- to 15-year period, with age being the most significant risk factor [1]. Visual impairment in the elderly negatively affects quality of life and increases the need for care because of increased fall risk, loss of independence, depression, and increased all-cause mortality [3–8]. A large longitudinal observational study in American adults concluded that regular eye examinations for those aged 65 or older are a protective factor for the development of decline in both vision and functional status [9]. This link between receipt of care and visual and functional outcome reinforces the current professional guidelines by the American Academy of Ophthalmology (AAO), which advocate a complete eye exam with an ophthalmologist every year or two after the age of 65 [10]. When it comes to screening for impaired visual acuity in elderly, however, current evidence appears to be insufficient to assess the balance of benefits and harms, as concluded by the US Preventive Services Task Force. The reason for these findings is the lack of well-designed studies demonstrating conclusive benefits of universal eye screening in the elderly [11].

Furthermore, glaucoma, and to a lesser extent AMD and DRP, are characterized by irreversible damage and vision loss, emphasizing the need for early diagnosis and treatment to delay the development of significant visual impairment. More than half of glaucoma cases remain undiagnosed, even in developed regions [12–15], despite widely available eye care facilities [16–18]. Glaucoma screening remains controversial because of the lack of data, economic evaluations, and accurate screening test algorithms [19–21], and diagnosis is mostly made by routine opportunistic case finding as there is no evidence for a useful screening tool to date. Nevertheless, the debate is ongoing and more evidence could argue for targeted screening or even for mass screening for glaucoma if more effective diagnostic tools become available. On the other end of the spectrum, glaucoma overdiagnosis and overtreatment is a relevant health issue, as pointed out by various authors and reviewed by González-Martín-Moro and Zarallo-Gallardo [12, 17, 22–25].

This study investigates the prevalence of age-related eye diseases in a cohort of self-proclaimed healthy elderly to get a rough estimation of the prevalence of undiagnosed age-related eye conditions in the Belgian population. The results underline the potential benefit of screening for a subset of prevalent sight-threatening age-related eye diseases in an elderly population, preferentially before the onset of impactful visual decline.

## 2. Materials and Methods

### 2.1. Study Design

This single-center cross-sectional study took place at University Hospitals UZ Leuven, Department of Ophthalmology, Leuven, Belgium, during April 2017. Elderly individuals free of known ophthalmological diseases were recruited from the members of the Third Age University Leuven, a KU Leuven initiative that offers a continued education program to the over-55-year-olds.

Those who received the advice of referral to an ophthalmologist or the advice to have more frequent follow-up visits, based on their participation in this study, received a questionnaire regarding their follow-up status in November 2018 (18 months after study participation).

Individuals with a known ophthalmological condition, besides refractive error or pseudophakia, and those with subjectively suboptimal visual acuity were excluded.

### 2.2. Study Population and Research Methods

The cohort included 102 people and comprised 46% men (median age 70 years, range 57–85 years). Inclusion criteria were members of the Third Age University Leuven (thus aged ≥55 years), with self-proclaimed healthy eyes and good general health. Written informed consent was obtained from each volunteer prior to inclusion in the study in compliance with relevant regulation on clinical trials. Individual results were discussed with each participant. In the case of an abnormal result, the participant was referred for further diagnostic work-up or regular follow-up.

### 2.3. Ophthalmological Examination

Subjects were asked to fill in a questionnaire on their personal and familial general and ocular history. A basic ophthalmological examination of both eyes of each participant was performed, including biomicroscopy, intraocular pressure (IOP) measurement by rebound tonometry using an iCare® TA01i tonometer (Tiolat Oy, Helsinki, Finland), axial length (AXL) measurement by IOL Master 700 (Carl Zeiss Meditec AG, Jena, Germany), dilated fundoscopy, and stereoscopic optic disc photography as well as macula-centered fundus photography using the Visucam PRO NM (Carl Zeiss Meditec AG, Jena, Germany) and optical coherence tomography (OCT) using the glaucoma module of the OCT Spectralis (Heidelberg Engineering, Heidelberg, Germany). The subset of volunteers that received advice to have further exams or more frequent follow-up received a questionnaire concerning their follow-up status where they were asked to answer questions about ophthalmologist visits since participating in this study and ocular interventions. All data were anonymized prior to analysis.

### 2.4. Statistical Analysis

Statistical analyses were performed using IBM SPSS® 25.0 for Windows (IBM, Armonk, New York, USA). Continuous variables were tested for normality using the Shapiro–Wilk test. Continuous variables are presented as the median, minimum, and maximum because they were not normally distributed (*P* < 0.05). Binary variables are presented as numbers and percentages. Nominal variables are presented as numbers with percentages per category. To statistically compare variables between groups, the Mann–Whitney *U* test was used for continuous variables and the chi-square test was used for dichotomous and nominal variables. Pairwise correlation was additionally assessed using Spearman's rank correlation coefficients. The influence of age, gender, education, smoking status, diabetes, arterial hypertension, neurological pathology, autoimmune pathology, intraocular lens status, familial history of glaucoma, familial history of AMD, intake of vitamin preparations for the prevention of AMD, the presence of a corrected refractive error, and previous ophthalmologist visits on the need for referral to an ophthalmologist was studied. Statistical significance was accepted based on two-sided *P* values of <0.05.

### 2.5. Compliance

The study was conducted in compliance with the principles of the European Union Directive on Clinical Trials (2001/20/EC) and all local/regional requirements required to conform with the provisions of the Declaration of Helsinki (World Medical Association, Edinburgh, 2000). Approval was issued by the Ethics Committee of the University Hospitals Leuven before the study commenced.

## 3. Results

### 3.1. Patients' Characteristics

The cohort included 102 people, who were all included for further analysis. Thus, 102 subjects and 203 eyes (one visitor was monophthalmic due to trauma) remained in the study group. Detailed baseline characteristics are listed in Table 1. The age ranged from 57 to 85 years (median 69.50 years) with 83% of the study population being aged ≥65 years. Overall, the subjects included were highly educated, with significantly more men reporting more than three years of higher education (*P* < 0.05). All subjects that had been diagnosed with systemic pathology stated that this pathology was well controlled and under follow-up. In accordance with the inclusion criteria, the ocular status was deemed healthy by all 102 volunteers, with the absence of subjective visual impairment.

**Figure 1:**
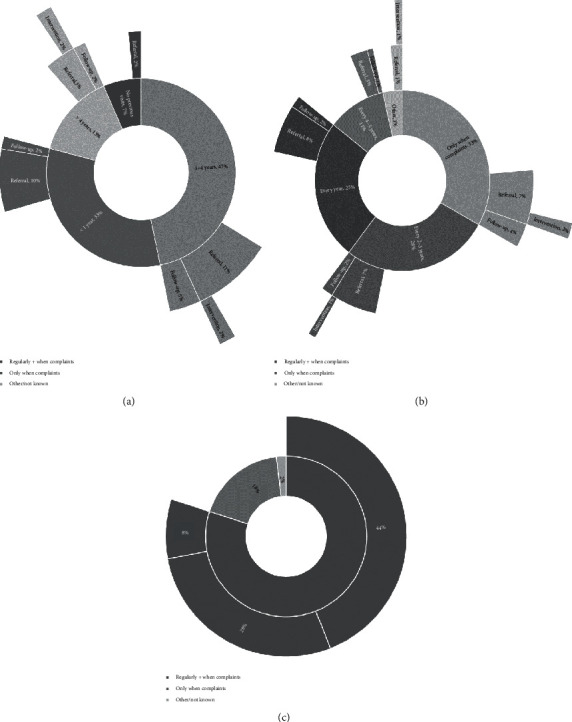
(a) Referral, follow-up, and intervention versus time since last eye doctor visit. (b) Referral, follow-up, and intervention versus eye doctor visits. (c) Eye doctor visits as recommended by volunteers.

Information regarding eye care consumption in this study cohort is depicted in Figures 1(a)–1(c). Before participating in this study, only 7 out of these 102 volunteers never consulted an ophthalmologist. Almost half of the study volunteers paid their ophthalmologist the last visit one to four years before taking part in this study. 64% reported regular eye doctor visits, while one-third only visited an ophthalmologist in case of complaints. On the other hand, 80% would recommend regular eye doctor visits, most of them advocating a visit interval of two to three years.

### 3.2. Clinical Results

The axial length ranged from 20.89 mm to 28.54 mm (median 23.69 mm). The intraocular pressure ranged from 8.00 mmHg to 35.00 mmHg (median 14.00 mmHg), and the cup-to-disc ratio from 0.10 to 0.85 (median 0.40).

**Figure 2:**
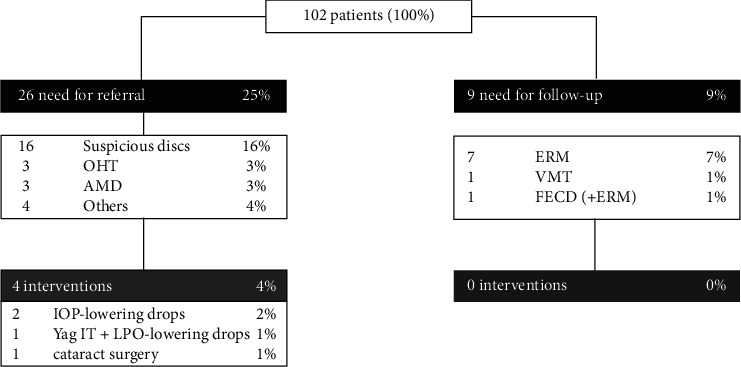
Flowchart. OHT = ocular hypertension; AMD = age-related macular degeneration; ERM = epiretinal membrane; VMT = vitreomacular traction; FECD = Fuchs' endothelial corneal dystrophy; IOP = intraocular pressure; IT = iridotomy.

As shown in Figure 2, 26 participants (25%) were referred for additional examinations based on the clinical findings. Three out of four referrals were due to signs of glaucomatous pathology. In 16 cases, suspicious optic discs were the reason for referral. Three participants had ocular hypertension, and two were referred due to signs of AMD (macular drusen). Other signs that led to referrals for additional examinations were episcleral/retinal vessel tortuosity, unspecific macular changes, cataract, and posterior capsule opacification. No cases of diabetic retinopathy or exudative AMD were observed. The advice to have more regular follow-up ophthalmologist visits was given to nine additional participants (9%) because of physiological findings which have the potential to become pathological over time. In seven cases, an asymptomatic epiretinal membrane (ERM) was the rationale behind follow-up advice. One participant's retina showed asymptomatic vitreomacular traction (VMT), and one asymptomatic volunteer presented with ERM as well as early signs of Fuchs' endothelial corneal dystrophy (FECD). This resulted in 35 participants (34%) selected for evaluation of follow-up status. Follow-up information was available for 25 out of 26 referred volunteers (96%) and for seven out of nine volunteers with only follow-up indicated (78%). Out of the former 25, four (16%) underwent a therapeutic intervention based on study referral: three were put on topical IOP-lowering drops, one participant underwent a Yag laser iridotomy as well, and one underwent cataract surgery. In the follow-up only group, no interventions have been recorded until 18 months after study participation.

The relation between eye care consumption and detected pathology is depicted in Figures 1(a)–1(c). In this small cohort, no significant correlations between baseline characteristics, including eye care consumption, and the need for referral could be identified.

Considering different age groups, relatively more referrals for additional eye examinations were made among the youngest participants (17% of participants), since 41% of those under 65 received the advice for additional exams as opposed to 19% of those between 65 and 74 years. A possible explanation for this finding is that many of those aged 65–74, willing to participate, and already had been diagnosed with an age-related eye disease by their ophthalmologist prior to this study, hence not meeting the inclusion criteria. All four interventions took place in the age group 65–74 years. The reasons for referral were equally distributed over the different age groups, except for the three cases of OHT, all occurring in the 65–74 years subgroup (Table 2).

## 4. Discussion

The aim of the current study was to investigate the prevalence of age-related eye diseases in a cohort of self-proclaimed healthy elderly and thus get a very rough estimation of the prevalence of undiagnosed age-related eye conditions in the Belgian population. This study shows that, even in a highly educated, self-conscious elderly cohort without visual complaints, potentially sight-threatening ocular pathology is detected, and therapeutic interventions are indicated in a significant proportion of individuals. Because of different sample characteristics, these results do not reflect the findings regarding prevalence and causes of visual impairment in a sample of the general population aged over 55, as has been extensively studied in large cohorts [26–35]. As the cohort studied here consists of participants of a continued education program, it is inherently biased by a societal interest, self-awareness of one's own health, and easier access to healthcare, which probably leads to an underestimation of the actual prevalence in the general population aged 55 years and older. The outcomes of the current study do not allow to draw population-wide conclusions, but they definitely consolidate the generally accepted and the AAO-formulated recommendation to visit an ophthalmologist every year or two from the age of 65 onwards [10]. Although the need for referral was relatively higher among those younger than 65 compared to the 65+ year group in this cohort (41% versus 22%, *P* > 0.05), all patients that needed an intervention belonged to the 65–74 years subgroup. A possible explanation for this finding is that many of those aged 65–74 and willing to participate, already had been diagnosed with, and potentially treated for an age-related eye disease by their ophthalmologist prior to this study, hence not meeting the inclusion criteria. As such, the latter age group might be considered as a particular target group for early diagnosis and initiation of treatment of age-related ocular pathology. Additionally, one in ten participants were advised to have regular check-ups with their ophthalmologist, bringing the total proportion of participants in whom additional examinations or more frequent follow-up were indicated to 34%. This means that one in three elderly could possibly benefit from regular ophthalmologist visits, which is in accordance with findings documented about 15 years ago by Sloan et al. [9]. However, the absence of interventions during the 18-month period after study participation among those advised to have a regular follow-up of identified changes might indicate that a larger interval between follow-ups suffices in certain conditions. For example, a short interval in cases of asymptomatic pathology that lacks therapeutic implications, such as ERM (with OCT), does not probably fit the cost-effective approach required by current medical care and healthcare systems.

The most prevalent causes of blindness and visual impairment are known to vary with age. A review by Finger and Scholl on blindness and visual impairment in high-income countries reported AMD, followed by DRP, as the most frequent causes of blindness and severe visual impairment in those aged 60–79 years. Above this age, AMD remained first, followed by glaucoma [1]. According to Klein and Klein, those aged 80 and older represent the heaviest burden of age-related eye disease in the United States, accounting for one-third of all cases of cataract, open-angle glaucoma, and early AMD and two-thirds of late AMD cases [36]. Due to its insidious nature, partly related to the centripetal pattern of visual field deterioration, glaucoma is expected to be more prominent in an asymptomatic cohort, such as the one studied here, compared to other prevalent and potentially sight-threatening age-related eye conditions such as AMD, often causing symptoms related to centrifugal central vision loss at an earlier disease stage.

Notwithstanding the fact that the majority of participants visited an ophthalmologist less than four years prior to the study, a considerable amount of potentially sight-threatening ocular conditions, which participants were not aware of, were uncovered by the comprehensive study protocol. This can be due to little importance attributed to the particular findings by the ophthalmologist, to findings missed by the ophthalmologist, or to the patients' inability to take in all information given during a consultation. As suggested by Keunen et al., in the Netherlands, more than half of the visually impaired aged 65 and above suffer from an eye disease that could have been treated or prevented, due to a large proportion of the elderly not making use of eye care [26]. This was confirmed by Cedrone et al. in Italy [27]. Seniors tend to believe that visual impairment is part of the aging process, making them less aware of gradual visual decline. No comparable data exist for the Belgian elders, but the present study concerns a subgroup of seniors with a certain degree of self-consciousness, in view of their willingness to participate in this study.

No high-risk groups for the development of visual impairment could be identified in the study cohort. This can be explained by a selection bias since patients that were already aware of having eye pathology were excluded. Additionally, the study population of overall healthy and mobile elders had easier access to health care than the average senior, and the majority of the study population paid regular visits to their ophthalmologist, where serious eye diseases had already been diagnosed. Nonetheless, there were several risk factors for visual impairment in this cohort, with aging being the most important one [29, 33, 37, 38]. There is no consensus on gender as a potential risk factor, but history of any ocular disease, diabetes mellitus, lower socioeconomic status, unemployment, and institutionalization have been reported as independent risk factors for visual impairment [26, 29, 33, 37, 38]. This shows once more that the average study participant had a low-risk profile for the development of visual impairment, except for age. It is striking, however, that even in such a “privileged” group, 26 referrals led to four interventions.

In this asymptomatic cohort, a low prevalence of clinically significant cataract, AMD, and DRP was expected. The pseudophakia prevalence of 9% in the current study corresponds well with German findings [39]. The single case with significant cataract in the current study was associated with long-term corticosteroid treatment, a well-known risk factor for the development of cataract [40]. The prevalence of asymptomatic macular drusen (1.96%) and focal VMT (0.98%) in the study population is in line with previous findings by Jacob and Stalmans in a sample of the Belgian population aged 34–66 years, taking into account the fact that the current study sample had a higher age [41]. Belgium has a prevalence of diabetes mellitus of 6.1% among its adults [42]. The vast majority of Belgian diabetic patients is older than 65 [43], resulting in a substantially higher prevalence among the elderly. A worldwide meta-analysis by Yau et al. revealed the presence of any form of diabetic retinopathy in 35.36% of diabetic patients [44], in line with the analysis of the National Health and Nutritional Examination Survey (NHANES) data noting a crude prevalence of diabetic retinopathy of 29.5% among patients with diabetes over 65 [45]. All this information, coupled with the fact that the current study population had a good general health and a considerably higher socioeconomic status, explains the low prevalence of diabetes mellitus and DRP [46].

Glaucomatous pathology is often characterized by a creeping onset and the lack of symptoms until extensive optic nerve damage has occurred. Together with a crude global prevalence of 3.54% in the population aged 40–80 years [47] and a risk that increases with age [15, 36, 47], glaucoma is a model example of an age-related, potentially sight threatening, ocular disease that can be detected in a cohort of asymptomatic elderly. According to a cross-sectional study by Shaikh et al., approximately 78% of US glaucoma cases are undetected and/or untreated, most of them being not even in their sixties [48]. This is in agreement with the three interventions related to glaucomatous diagnoses in the current study cohort. Comparable to the findings of Weih et al. [49], the Thessaloniki Eye Study proposed the lack of regular ophthalmologist visits as the main risk factor associated with undiagnosed open-angle glaucoma [13]. On the contrary, other authors stated that more than half of glaucoma patients identified by population screening had been previously examined by an ophthalmologist or eye care professional, and 17% even by an ophthalmologist in the two years prior to the screening visit [16]. In the current study, about half of the referrals and interventions were noted in the subgroup that paid regular visits to their ophthalmologist. All of the interventions and more than half of the referrals for additional examinations took place in participants who, if ever, last visited their ophthalmologist more than one year ago. This highlights the importance of the interval of ophthalmologist visits, but at the same time confirms that the detection threshold in the setting of study screening is different from that in clinical practice. This has been investigated by O'Neill et al. who showed that ophthalmology trainees as well as comprehensive ophthalmologists underestimated glaucoma likelihood in approximately one out of five-disc photographs and that they were twice as likely to underestimate as overestimate glaucoma likelihood, compared to glaucoma specialists [50]. The health system would not be able to cope with the burden of overreferral of “glaucoma suspects” with current management strategies. Perhaps virtual clinics and telemedicine, with or without the aid of advanced artificial intelligence algorithms that might arise in the near future, could allow examinations and follow-up to be performed to these “suspects” [51]. Only if progression, or other significant finding, was detected would they be sent back to the ophthalmologist. According to the US Preventive Services Task Force and the World Glaucoma Society, current evidence is insufficient to accept or reject the idea of routine glaucoma screening because of a shortage of data, regional economic evaluations, and screening test algorithms with high specificity. The question is raised whether targeted screening could be more effective [20, 21]. It will be crucial to determine the ideal window and tools to screen, ideally with a simple, reliable, and inexpensive screening test that can effectively detect disease at a time when intervention can have a significant impact on the patient's quality of life. This study suggests that screening for glaucoma and other age-related sight-threatening conditions could be worthwhile in (subgroups of) the 65–74 years old age group since all interventions took place in this age group of asymptomatic participants who are likely to have several more decades of living independently and in reasonably good health ahead of them. However, if screening for glaucoma and possibly other age-related ocular diseases such as cataracts, AMD, and DRP would be cost-effective, a clear strategy regarding referral, follow-up, and treatment needs to be defined since low referral uptake has also been demonstrated in the elderly [13, 52].

Limitations of the present study constitute first of all a selection bias, since those who participated may be more health conscious and take better care of themselves. This cohort will probably obtain more regular ophthalmologist visits than less health-conscious seniors and those belonging to disadvantaged communities with limited access to health care [53], but less than those with ocular conditions or vision problems and diabetes [54–56]. Secondly, some of the data were based on self-report, and no clinical measures of visual acuity have been made. A third limitation is the small sample size and, related to this, the small number of incident cases. Study strengths concern the absence of active ocular diagnoses and vision-related symptoms, both typical features of a senior population potentially suited for screening and the longitudinal component of this study. To our knowledge, this is the first study to assess the prevalence of various age-related ocular pathologies in a Belgian cohort of asymptomatic elderly. Studies in a larger cohort, more closely resembling the general population, and with a longer follow-up period would be required to take a position on policy recommendations. We believe that the recent Belgian measures that enable reimbursement of OCT acquisition for different age-related pathologies, such as AMD and glaucoma, could pave the way for future actions, meanwhile keeping in mind that keeping patients away from treatment also involves a cost of regular follow-up. Thus, future research focusing on the cost-effectiveness of regular eye examination versus screening programs is warranted. Visual impairment in the elderly is associated with many comorbidities, of which cognitive impairment is a very important one [57–59]. With the growing number of seniors in today's society, the risk of visual decline with aging needs to be effectively reduced to ensure healthy aging with preserved quality of life.

## 5. Conclusion

This study shows that, even in an elderly population with self-proclaimed healthy eyes and good general health, a significant proportion of subjects showed ocular findings that need regular follow-up and/or therapeutic intervention. Moreover, the frequency of prior ophthalmological examinations does not seem to be relevant to this proportion, meaning that everyone above 55 years could benefit from a routine ophthalmological evaluation.

## Figures and Tables

**Table 1:** Baseline characteristics of the study cohort.

Characteristics, *n* = 102	
Age (years), range (median)	57–85 (69.50)
Age (years in range), *n* (%)	
<65	17 (17)
65–74	64 (63)
75–84	20 (19)
≥85	1 (1)

Male sex, *n* (%)	47 (46)

Education, *n* (%)	
High school	12 (12)
≤3 years of higher education	66 (65)
>3 years of higher education	24 (23)

Smoking status, *n* (%), pack years, range (median)	
Current smoker	3 (3)–4.4–50.0 (28.0)
Former smoker	47 (46)–0.1–39.0 (8.5)
Never smoked	52 (51)

Diabetes, *n* (%)	8 (8)
Arterial hypertension, *n* (%)	39 (38)
Neurological disorder, *n* (%)	16 (16)
Autoimmune disorder, *n* (%)	14 (14)
Family history of glaucoma, *n* (%)	17 (17)
Family history of AMD, *n* (%)	6 (6)
Intake of vitamin preparations for the prevention of AMD, *n* (%)	3 (3)
Pseudophakia/aphakia in at least one eye, *n* (%)	9 (9)

Correcting spectacles, *n* (%)	
Distant sight	67 (66)
Reading	90 (88)

Time since last eye doctor visit, *n* (%)	
<1 year	34 (33)
1–4 years	48 (47)
>4 years	13 (13)
Never	7 (7)

AMD = age-related macular degeneration.

**Table 2:** Study findings according to the age group.

Age interval (years)	<65	65–74	75–84	≥85	Total
*n* (%)	17 (17%)	64 (63%)	20 (19%)	1 (1%)	102 (100%)

Need for referral, *n* (%)	7 (41%)	12 (19%)	7 (35%)	0 (0%)	26 (25%)

Reason for referral, *n*					
Suspicious discs	5	6	5	0	16
OHT	0	3	0	0	3
AMD	0	2	1	0	2
Others	2	1	1	0	5

Interventions, *n* (%)	0 (0%)	4 (29%)	0 (0%)	0 (0%)	4 (15%)

Need for follow-up, *n* (%)	2 (12%)	3 (5%)	3 (15%)	1 (100%)	9 (9%)

Reason for follow-up, *n*					
ERM	1	2	3	1	7
VMT	0	1	0	0	1
FECD	1 (+ERM)	0	0	0	1

OHT = ocular hypertension; AMD = age-related macular degeneration; ERM = epiretinal membrane; VMT = vitreomacular traction; FECD = Fuchs' endothelial corneal dystrophy.

## Data Availability

The data that support the findings of this study are available from the corresponding author upon reasonable request.

## Abbreviations

AMD: Age-related macular degeneration
DRP: Diabetic retinopathy
AAO: American Academy of Ophthalmology
IOP: Intraocular pressure
AXL: Axial length
OCT: Optical coherence tomography
ERM: Epiretinal membrane
VMT: Vitreomacular traction
FECD: Fuchs' endothelial corneal dystrophy
NHANES: National Health and Nutritional Examination Survey.

## References

[1] Finger R. P., Scholl H. P. (2013). Blindness and visual impairment: high-income countries. *Ophthalmology and the Ageing Society*.

[2] Flaxman S. R., Bourne R. R. A., Resnikoff S. (2017). Global causes of blindness and distance vision impairment 1990–2020: a systematic review and meta-analysis. *The Lancet Global Health*.

[3] Evans J. R., Fletcher A. E., Wormald R. P. L. (2007). Depression and anxiety in visually impaired older people. *Ophthalmology*.

[4] Lord S. R., Menz H. B., Sherrington C. (2006). Home environment risk factors for falls in older people and the efficacy of home modifications. *Age and Ageing*.

[5] Dhital A., Pey T., Stanford M. R. (2010). Visual loss and falls: a review. *Eye*.

[6] Senra H., Barbosa F., Ferreira P. (2015). Psychologic adjustment to irreversible vision loss in adults. *Ophthalmology*.

[7] Pedula K. L., Coleman A. L., Yu F. (2015). Age-related macular degeneration and mortality in older women: the study of osteoporotic fractures. *Journal of the American Geriatrics Society*.

[8] Soubrane G., Cruess A., Lotery A. (2007). Burden and health care resource utilization in neovascular age-related macular degeneration. *Archives of Ophthalmology*.

[9] Sloan F. A., Picone G., Brown D. S., Lee P. P. (2005). Longitudinal analysis of the relationship between regular eye examinations and changes in visual and functional status. *Journal of the American Geriatrics Society*.

[10] American Academy of Ophthalmology (2015). *Policy Statement-Frequency of Ocular Examinations San Francisco*.

[11] Siu A. L., Bibbins-Domingo K., Grossman D. C. (2016). Screening for impaired visual acuity in older adults: US preventive services task force recommendation statement. *Journal of the American Medical Association*.

[12] Cassard S. D., Quigley H. A., Gower E. W., Friedman D. S., Ramulu P. Y., Jampel H. D. (2012). Regional variations and trends in the prevalence of diagnosed glaucoma in the medicare population. *Ophthalmology*.

[13] Topouzis F., Coleman A. L., Harris A. (2008). Factors associated with undiagnosed open-angle glaucoma: the Thessaloniki eye study. *American Journal of Ophthalmology*.

[14] Mitchell P., Smith W., Attebo K., Healey P. R. (1996). Prevalence of open-angle glaucoma in Australia. *Ophthalmology*.

[15] Dielemans I., Vingerling J. R., Wolfs R. C. W., Hofman A., Grobbee D. E., de Jong P. T. V. M. (1994). The prevalence of primary open-angle glaucoma in a population-based study in the Netherlands. *Ophthalmology*.

[16] Grødum K., Heijl A., Bengtsson B. (2002). A comparison of glaucoma patients identified through mass screening and in routine clinical practice. *Acta Ophthalmol Scand*.

[17] Mukesh B. N., McCarty C. A., Rait J. L., Taylor H. R. (2002). Five-year incidence of open-angle glaucoma. *Ophthalmology*.

[18] Wong E. Y. H., Keeffe J. E., Rait J. L. (2004). Detection of undiagnosed glaucoma by eye health professionals. *Ophthalmology*.

[19] Tuulonen A. (2011). Cost-effectiveness of screening for open angle glaucoma in developed countries. *Indian Journal of Ophthalmology*.

[20] Weinreb R. N. (2008). *Glaucoma Screening*.

[21] Fleming C., Whitlock E. P., Beil T., Smit B., Harris R. P. (2005). Screening for primary open-angle glaucoma in the primary care setting: an update for the US preventive services task force. *The Annals of Family Medicine*.

[22] Founti P., Coleman A. L., Wilson M. R. (2018). Overdiagnosis of open-angle glaucoma in the general population: the Thessaloniki eye dtudy. *Acta Ophthalmologica*.

[23] González-Martín-Moro J., Zarallo-Gallardo J. (2016). Sobrediagnóstico y sobretratamiento en oftalmología: revisión de la literatura. *Archivos de la Sociedad Española de Oftalmología*.

[24] Fernández M. J., Leal M. Á., Guzmán J. (2010). Idoneidad de tratamiento en sospechosos de glaucoma. estudio de concordancia con el grupo de estudio RAND. *Archivos de la Sociedad Española de Oftalmología*.

[25] Zemba M., Cucu B., Stinghe A., Furedi G., Zugravu V., Lacusteanu M. (2008). Overdiagnosis in glaucoma-a real problem?. *Oftalmologia (Bucharest, Romania: 1990)*.

[26] Keunen J. E., Verezen C. A., Imhof S. M., van Rens G. H., Asselbergs M. B., Limburg J. J. (2011). Increase in the demand for eye-care services in the Netherlands 2010-2020. *Ned Tijdschr Geneeskd*.

[27] Cedrone C., Culasso F., Cesareo M. (2003). Incidence of blindness and low vision in a sample population. *Ophthalmology*.

[28] Congdon N., O’Colmain B., Klaver C. C (2004). Causes and prevalence of visual impairment among adults in the United States. *Archives of Ophthalmology (Chicago, Ill. :1960)*.

[29] Dimitrov P. N., Mukesh B. N., McCarty C. A., Taylor H. R. (2003). Five-year incidence of bilateral cause-specific visual impairment in the melbourne visual impairment project. *Investigative Opthalmology & Visual Science*.

[30] Finger R. P. (2007). Blindheit in Deutschland: dimensionen und perspektiven. *Der Ophthalmologe*.

[31] Finger R. P., Fimmers R., Holz F. G., Scholl H. P. N. (2011). Incidence of blindness and severe visual impairment in Germany: projections for 2030. *Investigative Opthalmology & Visual Science*.

[32] Finger R. P., Fimmers R., Holz F. G., Scholl H. P. N. (2011). Prevalence and causes of registered blindness in the largest federal state of Germany. *British Journal of Ophthalmology*.

[33] Foran S., Wang J. J., Mitchell P. (2003). Causes of visual impairment in two older population cross-sections: the blue mountains eye study. *Ophthalmic Epidemiology*.

[34] Huang S., Zheng Y., Foster P. J., Huang W., He M. (2009). Prevalence and causes of visual impairment in Chinese adults in urban southern China. *Archives of Ophthalmology*.

[35] Klein R., Klein B. E. K., Lee K. E., Cruickshanks K. J., Gangnon R. E. (2006). Changes in visual acuity in a population over a 15-year period: the beaver dam eye study. *American Journal of Ophthalmology*.

[36] Klein R., Klein B. E. (2013). The prevalence of age-related eye diseases and visual impairment in aging: current estimates. *Investigate Ophthalmology and Visual Science*.

[37] Klein R., Klein B. E. K., Lee K. E. (1996). Changes in visual acuity in a population. *Ophthalmology*.

[38] Yonekawa Y., Varma R., Choudhury F., Torres M., Azen S. P., Group L. A. L. E. S. (2011). Risk factors for four-year incident visual impairment and blindness: the Los Angeles Latino Eye Study. *Ophthalmology*.

[39] Schuster A. K., Pfeiffer N., Schulz A. (2017). The impact of pseudophakia on vision-related quality of life in the general population-the Gutenberg health study. *Aging*.

[40] Prokofyeva E., Wegener A., Zrenner E. (2013). Cataract prevalence and prevention in Europe: a literature review. *Acta Ophthalmologica*.

[41] Jacob J., Stalmans P. (2016). Prevalence of vitreoretinal interface abnormalities as detected by spectral-domain optical coherence tomography. *Ophthalmologica*.

[42] Federation International Diabetes https://www.idf.org/our-network/regions-members/europe/members/125-belgium.html.

[43] Wild S., Roglic G., Green A., Sicree R., King H. (2004). Global prevalence of diabetes: estimates for the year 2000 and projections for 2030. *Diabetes Care*.

[44] Yau J. W. Y., Rogers S. L., Kawasaki R. (2012). Global prevalence and major risk factors of diabetic retinopathy. *Diabetes Care*.

[45] Zhang X., Saaddine J. B., Chou C.-F. (2010). Prevalence of diabetic retinopathy in the United States, 2005-2008. *Journal of the American Medical Association*.

[46] Rabi D. M., Edwards A. L., Southern D. A. (2006). Association of socio-economic status with diabetes prevalence and utilization of diabetes care services. *BMC Health Services Research*.

[47] Tham Y.-C., Li X., Wong T. Y., Quigley H. A., Aung T., Cheng C.-Y. (2014). Global prevalence of glaucoma and projections of glaucoma burden through 2040. *Ophthalmology*.

[48] Shaikh Y., Yu F., Coleman A. L. (2014). Burden of undetected and untreated glaucoma in the United States. *American Journal of Ophthalmology*.

[49] Weih L., Nanjan M., McCarty C. A., Taylor H. R. (2001). Prevalence and predictors of open-angle glaucoma Results from the visual impairment project. *Ophthalmology*.

[50] O’Neill E. C., Gurria L. U., Pandav S. S. (2014). Glaucomatous optic neuropathy evaluation project: factors associated with underestimation of glaucoma likelihood. *JAMA Ophthalmology*.

[51] Olivia Li J. P., Liu H., Ting D. S. J. (2020). Digital technology, tele-medicine and artificial intelligence in ophthalmology: a global perspective. *Progress in Retinal and Eye Research*.

[52] van Nispen R., van der Aa H., Timmermans F. (2018). Reducing avoidable visual impairment in elderly home healthcare patients by basic ophthalmologic screening. *Acta Ophthalmologica*.

[53] Maberley D. A. L., Hollands H., Chang A., Adilman S., Chakraborti B., Kliever G. (2007). The prevalence of low vision and blindness in a Canadian inner city. *Eye*.

[54] Zhang X., Saaddine J. B., Lee P. P. (2007). Eye care in the United States. *Archives of Ophthalmology*.

[55] Puent B. D., Klein B. E. K., Klein R., Cruickshanks K. J., Nondahl D. M. (2005). Factors related to vision care in an older adult cohort. *Optometry and Vision Science*.

[56] Orr P., Barrón Y., Schein O. D., Rubin G. S., West S. K. (1999). Eye care utilization by older americans. *Ophthalmology*.

[57] Anstey K. J., Luszcz M. A., Sanchez L. (2001). Two-year decline in vision but not hearing is associated with memory decline in very old adults in a population-based sample. *Gerontology*.

[58] Lin M. Y., Gutierrez P. R., Stone K. L. (2004). Vision impairment and combined vision and hearing impairment predict cognitive and functional decline in older women. *Journal of the American Geriatrics Society*.

[59] Reyes-Ortiz C. A., Kuo Y.-F., DiNuzzo A. R., Ray L. A., Raji M. A., Markides K. S. (2005). Near vision impairment predicts cognitive decline: data from the hispanic established populations for epidemiologic studies of the elderly. *Journal of the American Geriatrics Society*.

